# Analyzing EEG Signals Using Decision Trees: A Study of Modulation of Amplitude

**DOI:** 10.1155/2020/3598416

**Published:** 2020-07-09

**Authors:** Narusci S. Bastos, Bianca P. Marques, Diana F. Adamatti, Cleo Z. Billa

**Affiliations:** Federal University of Rio Grande, Computer Science Center, Rio Grande, RS 96203-900, Brazil

## Abstract

An electroencephalogram (EEG) is a test that records electrical activity of the brain using electrodes attached to the scalp, and it has recently been used in conjunction with BMI (Brain-Machine Interface). Currently, the analysis of the EEG is visual, using graphic tools such as topographic maps. However, this analysis can be very difficult, so in this work, we apply a methodology of EEG analysis through data mining to analyze two different band frequencies of the brain signals (full band and Beta band) during an experiment where visually impaired and sighted individuals recognize spatial objects through the sense of touch. In this paper, we present details of the proposed methodology and a case study using decision trees to analyze EEG signals from visually impaired and sighted individuals during the execution of a spatial ability activity. In our experiment, the hypothesis was that sighted individuals, even if they are blindfolded, use vision to identify objects and that visually impaired people use the sense of touch to identify the same objects.

## 1. Introduction

The human brain is a multifaceted structure that is capable of storing large amounts of information, transforming it, learning and making complex decisions, and providing us with the ability to discover and influence the world [1]. In this sense, neuroscience is an interdisciplinary science that joins different areas of knowledge with the intention of interpreting the nervous system as a whole. The BMI (Brain-Machine Interface) is an application that allows communication between an individual and an external device such as a computer without any muscle movements created by the brain [2]. These devices communicate directly through brain impulses, such as those captured in an EEG (electroencephalogram). The EEG is based on records of electrical brain activity that are measured on the surface of the scalp. These high-temporal-resolution systems are able to measure brain signals in milliseconds, generating a large amount of data.

There are different neuroscience studies based on BMI systems. Some of them study how to provide a better quality of life for people with severe motor problems [3], rehabilitation of stroke victims [2], robotic systems movement, and classification of teaching objects related to the students level of attention [4]. The most commonly practised application of EEG is to monitor and study EEG records visually [5]. Graphical analysis can be very useful, but sometimes it is not sufficient because the amount of data is too large and thus can be very difficult to analyze [6].

To facilitate this process, this work uses a data-mining-based methodology to analyze EEG signals [7]; more specifically, we use decision trees algorithms to analyze brain signals. Decision trees are very easy to read, and through them it is possible to understand which area of the brain was activated or how many times a pattern occurred [8].

To show how the methodology works, we present a case study that exploits the spatial ability of visually impaired and sighted people. Both groups had to identify spatial objects in a controlled environment. Our hypothesis is that each group would use different brain areas to perform such activities because visually impaired individuals tend to compensate for the sense of sight with other senses [9].

A BMI-EEG, typically, records brain signals in the range of 0.1 Hz to 100 Hz. Wave forms are subdivided into bandwidths known as Alpha, Beta, Theta, and Delta to signify the majority of the EEG [5]. In this work, we are particularly interested in analyzing the Beta frequency band because it is associated attention, visual precision, and coordination states.

In this work, we compare the full band frequency and the Beta band frequency used as input of two decision tree algorithms, J48 and Random Tree. We have two goals in this paper: firstly, we present the complete proposed methodology, initially presented in [7], and all analysis of the data to validate our hypothesis; secondly, we analyze that there is a best algorithm and a best input band frequency to use data-mining-based methodology to analyze EEG signals.

This paper is organized as follows.  Section 2 presents the theoretical background used for the development of the methodology and the case study.  Section 3 shows the proposed methodology used. In Section 4, the first case study and the generated models (decision trees) are presented.  Section 5 presents the second case study that uses modulation of amplitude. Finally, in Section 6, we discuss the results of this research.

## 2. Theoretical Basis

### 2.1. Brain and Actions

The brain is the main component of the nervous system. It is responsible for all mental operations, such as concentration, thinking, learning, and motor control. These capabilities are implemented through neurons in ways that can currently be explained by neuroscience.

The human brain is divided into two hemispheres, right and left. Initially, there was a belief that there was one dominant hemisphere, and the other was dominated. However, this concept has become outdated, and now there is a belief that there are actually two specialized hemispheres. Thus, each hemisphere is responsible for a set of functions that ultimately work together. Anatomists usually divide the brain into major regions, called lobes, whose boundaries are not always precise but transmit an initial idea of regional location. There are five lobes: four external and one internal, located in the lateral sulcus [10]. The four external lobes are the frontal lobe, which is located in the forehead; the parietal lobe, which is located under the cranial bone of the same name; the temporal lobe, which is associated with the temple; and the occipital lobe, which is located in the occipital cranial bone. The fifth lobe, the insular lobe, can only be seen when the lateral sulcus is opened [10, 11]. There are many other structures situated in the central nervous system (CNS), but in this work we investigate only the four visible lobes because the BMI system that we used does not have access to the insula lobe.

Each lobe has specialized functions. The occipital lobe is primarily concerned with the sense of vision, and it is divided into multiple distinct visual areas, the largest of which is the primary visual cortex. The parietal lobe is partially dedicated to the sense of touch and is responsible for body sensitivity functions and spatial recognition. The temporal lobe contains the primary auditory cortex; it processes audio data, language understanding, specific aspects of vision, and some aspects of memory. Finally, the frontal lobe is responsible for cognitive actions, memory, and movement [10, 11].  Table 1 presents the brain areas and their main functions.

### 2.2. Visual Impairment

Visual impairment to any degree impairs a person's ability to orient themselves and move in space with security and independence [12]. An individual may be born without visual capacity or lose it during their life. Because spatial information does not reach a visually impaired individual through vision, he or she perceives the world using other senses: hearing, smell, touch, and taste [9].

Among the senses, hearing is extremely important because it is through hearing that that which cannot be seen can be interpreted (understood) through language, for example, helping a child with visual impairment to understand that there is a separate external reality [9, 12]. However, it is necessary that sighted individuals describe what is visual; thus [9] reminds us that sighted individuals are less accustomed to perceiving the world through the other senses, which requires the visually impaired individuals to make frequent “adjustments” to what s/he knows through her/his perceptions and what s/he knows through speech from those who are around him/her. In this context [13] states that “some visually impaired individuals become very sensitive to the nuances of inflection, volume, resonance and various intensities of speech sounds of others that go unnoticed to sighted individuals.” This does not mean that the visually impaired have a super capacity but rather that they use in-depth hearing [9].

Hearing also plays an important role in differentiating stimuli and detecting obstacles, as in the echolocation phenomenon. Through the location of objects based on sounds that are often not heard by sighted individuals, hearing may provide the visually impaired with indications of the direction and distance of objects [14]. To [14], touch is considered the most appropriate way to provide the displacement of reference in space that has left or never existed due to lack of vision, and it is through this that most of the knowledge space should be built; [15], in his findings, states that “identification through touching objects is not simply done by touching them and exploring them, it is necessary to develop a tactile sensitivity to them and/or know them.”

Other senses, such as taste and smell, also contribute to the expansion of visually impaired individuals' knowledge of their local living spaces and social relations and their day to day activities. The odors of where they have been provide possible knowledge of the objects that make up the place [15]. Smell helps people, visually impaired or not, realize, (re)know, and study the various objects that make up the landscape of a site, whether natural or artificial.

However, taste can provide a sense of tasting flavors with or without food and drink. According to [15], taste stimulates socialization when conducting meetings in environments where food is present, such as restaurants.

In this context, the use of each of the senses should not be considered separately. In [16], identification of a held object was much more efficient if the individual used the largest possible number of methods for the recognition rather than a single method.

### 2.3. BMI (Brain-Machine Interface) Systems

BMI systems consist of tools that enable communication based on neural activity generated by the brain without a need for any other type of brain signals, such as muscle movement. These use electrical signals detected on the scalp of the surface cortical or subcortical areas. The goal of BMIs is to provide interaction between the user and an external device, such as a computer, switch, or prosthesis [2].

One of the most commonly used techniques for capturing neural activity in BMI systems is EEG. The EEG is based on brain electrical activity records performed through electrodes applied to the scalp.

The signals that are captured by the EEG equipment measure potential differences between regions of the cortex. These differences are due to the flow of ions between different neurons in the brain. When the neuron is activated, it polarizes, generating an action potential that can be propagated to other neurons, thus generating the flow of information. Thus, EEG records, collected on the surface of the scalp, show the electrical activity of the brain [17].

The records obtained through the electrodes present the intensity of brain waves, which can vary between 0 and 200 *νµ* frequency bands, ranging from 0.3 Hz to 100 Hz.

The resulting signal of an EEG shows peaks related to the existence of electrical activity, showing a very general spatial location of this activity because this signal is the sum of the activity of a large number of neurons that communicate with each other [18].

### 2.4. Brain Waves

Brain waves can be classified using their frequency, amplitude, shape, and electrodes position on the scalp. EEG applications focus on a narrow band, from 0.1 Hz to 100 Hz. EEG signals are classified based on their frequency band [5].

- Delta waves (*δ*): They are in frequency band from 0.5 Hz to 3.5 Hz. They are slower than the others, and they generally occur in deep sleep and, sometimes, when in mental comma state.
- Theta waves (*θ*): They are in the frequency band from 3.5 Hz to 7.5 Hz. They appear during creative thought, stress, and deep meditating state.
- Alpha waves (*α*): They are in frequency band from 7.5 Hz to 12 Hz. They dominated calm and relaxed mental states.
- Beta waves (*β*): They are in the frequency band from 13 Hz to 30 Hz, and they are associated with attention, visual precision, and coordination states.
- Gamma waves (*γ*): They have frequency higher than 30 Hz. Motor functions, simultaneous work, and other multitasking occur in this range of frequency.

### 2.5. Decision Trees

The decision tree model is a supervised classification technique based on the division of a complex problem into several subproblems, repeating this process recursively to generate a tree. In a decision tree, each leaf node receives a class label; nonterminal nodes, which include the root node and other internal nodes, contain attribute testing conditions to separate records that have different characteristics [6].

In the late 1970s and early 1980s, J. Ross Quinlan developed ID3 (Iterative Dichotomiser 3), an algorithm to generate a decision tree. Some years later, he proposed the C4.5 algorithm as an optimized version of ID3. According to [19], C4.5 serves as a basis for new supervised methods. The J48 algorithm is an extension of the C4.5 classification algorithm, arising from the need to recode the algorithm into the Java language because C4.5 was originally written in the C language [20]. The algorithm always uses the best locally evaluated step, without concern as to whether this step will produce the best solution, and it divides a problem into several subproblems by creating subtrees between the root and the leaves [21].

In general, the classification algorithms aim to search for models that reach the highest precision or the lowest error rate when applied to the test set.

Random Tree is a randomly induced tree from a set of possible trees, using *m* random attributes on each node; each tree has an equal chance to be sampled, and it can be generated efficiently, and the combination of large sets of Random Trees usually leads to accurate models [22].

## 3. Proposed Methodology

**Figure 1:**
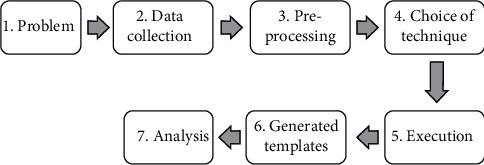
Proposed methodology in this work. In each step a sequence of actions is performed.

This paper aims to present a methodology for the use of decision trees as a means to understand brain activity from a case study with the recognition of spatial objects. Thus, seven steps are proposed, as presented in Figure 1.

- Problem: This stage consists of defining the problem or hypothesis to investigate its truth.
- Data collection: There are different methods of measuring brain activity, one of which is electroencephalography. This step consists of defining the tools and protocols for the collection of brain signals.
- Preprocessing: In this stage, the collected EEG data undergo a series of processes that allow the use of software for data mining (DM).
- Choice of technique: The choice of a technique means choosing the DM algorithm that best applies to the problem.
- Execution: This step refers to the definition of the parameters that must be used for the application of the DM algorithm.
- Generated templates: MD tools allow researchers to view extracted patterns or models that summarize the structure and information in the data.
- Analysis: The results obtained through the generated models are analyzed and validated. For this, it is important to include specialists in the field of study to validate and guarantee the consistency of the results.

## 4. First Case Study: Identification of Geometric Objects

Our study applies a decision tree as a means of providing EEG signal analysis. In this case study, the methodology proposed in Section 3 is applied for the EEG analysis of congenitally blind individuals and individuals with regular vision during the activity of identifying geometric objects. Next, the seven steps are applied within the proposed scenario.

### 4.1. Problem

For people without visual impairment, spatial analysis tasks, such as the identification of objects or people and location and movement in space, are naturally dominated by the sense of sight. Visual signals are sent to and processed in the occipital lobe. Therefore, people who are born without vision or lose their vision over time have a compromised visual sense, and they need to use other senses as a way to overcome the obstacle of the absence of vision. In [14, 16], touch is considered to be the most appropriate sense to provide displacement references in space. This sense is processed in the parietal lobe.

According to [23], “there is a common perceptual system to both the tactile and the visual sense: a sighted person uses a combination of sense, but in a blind person, they access only the tactile sphere.”

This case study's research question is as follows: do sighted people and visually impaired people use different brain areas when the spatial ability is activated?

The hypothesis is that sighted people and visually impaired people use different areas of the brain to “visualize” spatial objects. In sighted people, primarily the occipital lobe is activated. In the visually impaired people, primarily the parietal lobe is activated.

### 4.2. Data Collection

To perform this case study, the brain signals of 4 individuals, 2 sighted and 2 visually impaired, were collected. The individuals were asked to identify different 3D solid geometric shapes to activate their spatial abilities. There were three different objects: cube, rectangular prism, and pyramid.

All tests were performed with the approval of CEPAS (Research Ethics Committee at the Health Area in Brazil—CCAAE: 344172114.3.0000.5324), which provides models of documents and standards that must be followed. Some of these documents include the form of referral, a model of the TCLE (informed consent terms), and the terms of the commitment for the use of the data.

All experiments were performed in accordance with relevant guidelines and regulations. And all participants signed the informed consent form.

#### 4.2.1. Tools Used for the Acquisition of Brain Signals

**Figure 2:**
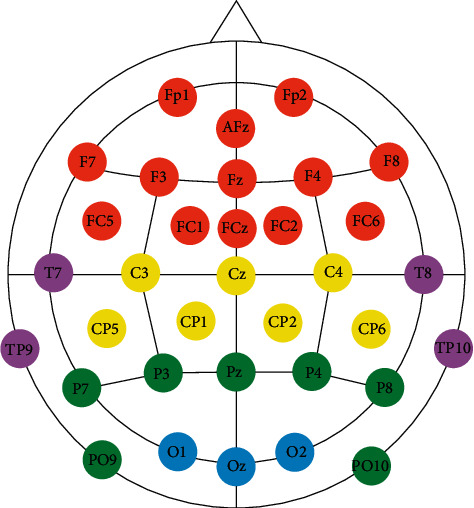
Location of the electrodes in the actiCAP, according to the international 10–20 system.

**Figure 3:**
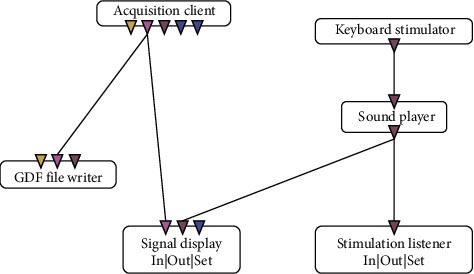
Automaton used for recording and monitoring brain signals.

- actiCHamp: The actiCHamp tool was developed by Brain Vision, LLC (https://brainvision.com/). It is a modular amplification system that incorporates large components for electrophysiological analysis, such as EEG, event-related brain potentials (ERP), and BMI. It was used in conjunction with actiCAP, which is a cap with 32 electrodes that is placed over the persons scalp. The actiCAP uses the channels of the international “10–20” system. The actiCAP is connected to the actiCHamp amplifier to transmit the signals captured by the electrodes.  Figure 2 shows how the electrodes are distributed throughout the cap.  Figure 2 represents the international “10–20” system, with 35 electrodes, but the actiCAP equipment is slightly different since it has only 32 electrodes. Electrodes Cz, C3, and C4 are missing on the actiCAP equipment. The location of each electrode is calculated to be at the intersection of the lines between the standard cranium landmarks, as shown in Figure 2. The name of each electrode indicates the corresponding region of the brain: FP indicates the prefrontal lobe; *F*, frontal lobe; *T*, temporal lobe; *C*, the central groove; *P*, parietal lobe; and O, occipital lobe. The number or the second letter identifies the hemispheric location: *Z* is the zero line in the center of the head; even numbers represent the right hemisphere; odd numbers represent the left hemisphere. The numbers are displayed in ascending order with increasing distance from the center [24, 25].
- OpenViBE: OpenViBE is a software platform dedicated to designing, testing, and using BMI systems. The configuration for use with actiCHamp is predefined, and the software communicates automatically with the signal capturing tool. OpenViBE presents a very simple interface in which the user can set the features that meet the needs of the task through an algorithm (automaton).  Figure 3 shows the scenario created for the acquisition of the brain signals and visual monitoring of the same; the automaton that follows the left side ending in the “signal display” is performed only at the level of monitoring tests, and, commenting further, the algorithms do not interfere with the signal acquisition. The data were recorded in GDF format (for each of the 32 electrodes, a column with the signal values is stored), and the preprocessing stage was then performed to enable the use of the same data in other tools. Each box of the automaton is explained below:
- Acquisition client box allows the acquisition of signals through the actiCAP electrodes.
- GDF file writer records the values of the electrical signals in GDF format.
- Signal display allows the brain signals to be followed through a wave chart.
- Keyboard simulator allows you to select ON/OFF keys.
- Sound player emits a sound signals according to the command of the keyboard signal (01—ON; 02—OFF).
- Stimulation listener records the events performed; in this case, it marks the times at which each sound signal was performed.

### 4.3. Collecting Brain Signals

1. Brain signals were collected from 2 sighted and 2 visually impaired individuals.
2. The cap was placed on the subject's head, where the 32 actiCAP electrodes were coupled.
3. Start of the collection stage: This stage consisted of the signals of each electrode reaching impedance necessary to perform the collection of the signals. The actiCAP electrodes allow visualization through a colored light that varies between yellow: low; orange: medium; and green: good. In addition, it offers a panel that presents the values of this impedance.
4. The person was instructed to feel and identify the object that was placed in her/his hand at the end of a sound signal. For the object identification, three objects of geometric shapes were made available: cube, rectangular prism, and pyramid. Six repetitions were performed, in which the subject probed and identified the objects 18 times. The interval between the repetitions was, on average, 10 minutes, and each repetition contained a sequence different from the previous order of the objects.
5. When the object name was verbalized, step (4) was executed again.

### 4.4. Preprocessing

The data went through four stages of preprocessing, as follows.

#### 4.4.1. Conversion from GDF File to CSV File

The signals were recorded with OpenViBE, without the use of filters, directly as GDF (graph data format). The filters were applied when the data were translated into CSV (comma-separated value) format. For the data conversion in OpenViBE, an algorithm was used to transform GDF data to CSV data, and a filter was applied. Preprocessing was performed for a better reading of the data.

#### 4.4.2. Data Labeling

Each line of the CSV file was labeled with the objects used in the collection, that is, the cube, the rectangular prism, or the pyramid, because the decision tree is a supervised algorithm. Through the analysis of the records of the sonorous signal start and end of the collection, each time interval was labeled according to the subject that was analyzed and identified by the person.

#### 4.4.3. Data Cleaning

After labeling the data, columns and lines that were not required for the application of the technique were removed. The column for time and the line for the channels were removed.

#### 4.4.4. Transformation of CSV Data to ARFF

The data were adapted to the ARFF format, which is the standard Weka (https://www.cs.waikato.ac.nz/ml/weka) format. This format describes a list of instances that share a set of attributes. This format was developed to be used specifically in Weka. In summary, the ARFF files have two different sections: the header, which contains the names of the relations along with a list of attributes and their types, and information data.

### 4.5. Definition of the Technique

The J48 algorithm was chosen because it is a decision tree technique developed to be used in Weka. The decision tree technique has, as its strong point, efficiency of time and processing, in addition to presenting an intuitive means of analyzing the results, because it shows, as the final structure of the classifier, a simple symbolic representation that is usually easy to interpret, facilitating the understanding of the problem under analysis [26]. The ease and the form of the symbolic representation were fundamental factors for the choice of this technique because the work is aimed at understanding the data through the decision tree and not just the efficiency of the classification.

### 4.6. Execution of the Technique

From the data recorded, different tests were performed using the decision trees. These tests were based on the variation of the following parameters: number of channels, signal values, and the order of the execution of the tasks.

The test used for the final analysis presents the models generated from the grouping of the data, with the highest values of each group for each of the 32 channels. Six models were generated for each sample, using windows (the number of values to maximize) of 25, 50, or 100 values, varying the percentage of the minimum number of objects of the leaf (1% or 10%).

### 4.7. Generated Templates

From the application of the methodology proposed in Section 3, decision tree models were generated for each of the four people. Persons 1 and 2 are sighted, and Persons 3 and 4 are congenitally visually impaired.

#### 4.7.1. Person 1

**Figure 4:**
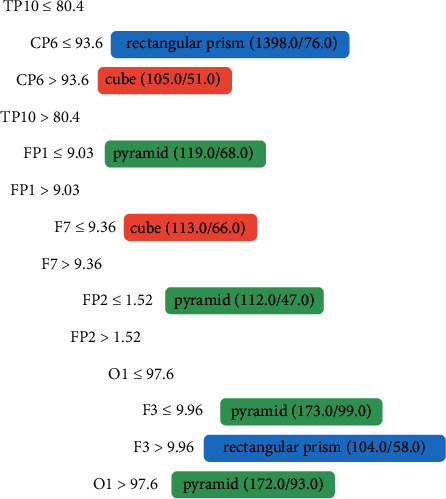
Tree generated from algorithm J48 referring to the data of Person 1, a sighted person.

The channels that presented significant activity in the model shown in Figure 4 were TP10, CP6, FP1, F7, FP2, O1, and F3. The channels FP1, F7, FP2, and F3 correspond to the frontal lobe, as presented in Table 1, and this area is responsible for the executive functions and management of cognitive resources. The TP10 channel belongs to the temporal lobe, which addresses the perception of biological movements. The CP6 channel belongs to the parietal lobe and is responsible for the tactile sense. The O1 channel belongs to the occipital lobe where the image processing takes place.

#### 4.7.2. Person 2

**Figure 5:**
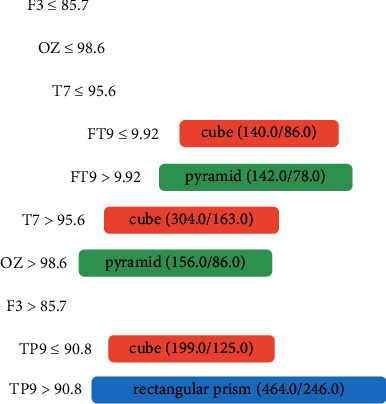
Tree generated from algorithm J48 referring to the data of Person 2, a sighted person.

Figure 5 shows the relevant activity channels F3, OZ, T7, FT9, and TP9. The frontal lobe channels F3 and FT9 are responsible for cognitive functions and reasoning; T7 and TP9 are in the temporal lobe, which is responsible for the perception of biological movements; and the occipital lobe OZ channel involves visual perception as well as the recognition of objects.

#### 4.7.3. Person 3

**Figure 6:**
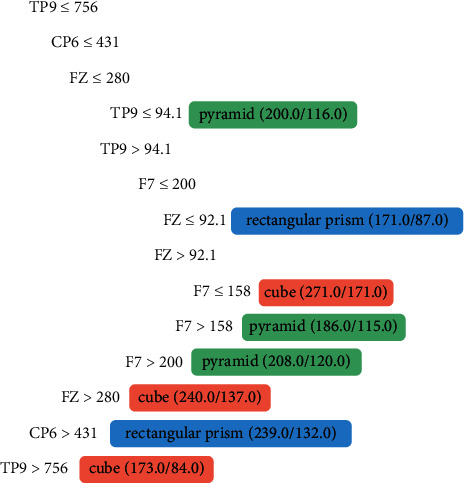
Tree generated from algorithm J48 from the data of Person 3, a congenitally visually impaired person.

The channels with relevant activity (in Figure 6) were TP9 and T7 in the temporal lobe, FZ and F7 in the frontal lobe, and CP6 in the parietal lobe. The analysis performed in Table 1 shows that the main functions involved in object recognition are perception of biological movements (temporal lobe, TP9 and T7); tactile sensation (parietal lobe, CP6); and the frontal area (FZ, F7), responsible for decision-making and movement planning.

#### 4.7.4. Person 4

**Figure 7:**
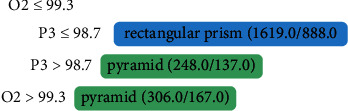
Tree generated from algorithm J48 from the data of Person 4, a congenitally visually impaired person.

In the tree generated for Person 4 (Figure 7), who is congenitally visually impaired, significant activity is observed in channels O2 and P3. These channels involve two large areas, the occipital lobe (O2), which is responsible for visual ability, and the parietal lobe (P3), which is used for tactile function.

#### 4.7.5. Data Analysis

According to [23], there is a common perceptual system shared between the tactile and visual sense, in which individuals with normal vision can achieve insights from a combination of senses that are accessible to the visually impaired only from tactile information. Therefore, the hypothesis of this work is that visually impaired and sighted people use different areas of the brain to “visualize” spatial objects. Sighted people use the occipital lobe, which is responsible for visualization, and visually impaired people primarily use the parietal lobe, an area responsible for tactile perception.

To verify the hypothesis in question, we performed tests using a decision tree with the J48 algorithm in the DM software Weka. The trees generated through the execution of the J48 algorithm did not present high accuracy rates (45% in average—see Table 2) in a classification task. However, the main goal of this work was not to classify the data; it is important to note that, for the analysis of the case studies in this work, the higher rate was taken into account for the selection of the trees that were presented. However, the accuracy rate is not a determinant for the understanding of the electrode paths because the intention is to analyze the generated trees and to understand the brain activities during the identification of the geometric objects.

Table 3 presents a summary of the areas that had some channel in the tree branches generated for each person. Based on Table 3, we can see that the sighted individuals (Person 1 and Person 2) used the occipital lobe to identify spatial objects, supporting the hypothesis that sighted individuals activate the occipital lobe.

The visually impaired people (Person 3 and Person 4) activated electrodes that belong to the parietal lobe in the branches of the generated trees, an area that is responsible for the tactile perception, supporting the hypothesis that the congenitally visually impaired use the parietal lobe to identify the object.

However, Person 4 (congenitally visually impaired) activated the O2 (occipital lobe) channel in the generated tree. The relevance of the brain signals in the occipital area can be derived from the idea presented in [19] that the activation of the occipital cortex often reflects the processing of mental visual images triggered by other senses. Thus, the occipital lobe, during touch identification, may serve as the basis of cross-plasticity observed in congenitally visually impaired individuals.

## 5. Second Case Study: Modulation of Amplitude

Using the same methodology and data collection, we have changed just three steps in relation to the first case study: the problem, the preprocessing, and the choice of technique.

**Figure 8:**
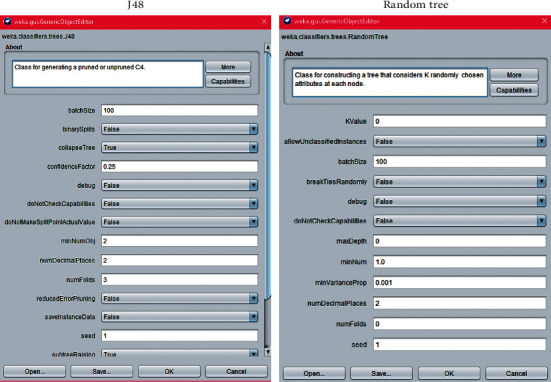
Configuration of J48 and Random Tree algorithms in Weka.

(i) Problem: testing the amplitude modulation (the Beta frequency band (*β*)) and discovering new results.
(ii) Two hypotheses were defined for this second case study, which will be compared to the results obtained in the first case study.
  - Random Tree has a higher classification accuracy than J48.
  - Both algorithms will get a higher classification accuracy using only the Beta frequency band.
(iii) Preprocessing: Using the complete data from first case study, we have used a filter to choose the Beta frequency band (13 Hz to 30 Hz) and the data were translated into CSV (comma-separated value) format.
(iv) Choice of technique: The choice of a technique means choosing the data-mining algorithm that best applies to the problem. In this case study, we have tested the data with two decision tree algorithms: J48 and Random Tree. The configuration of these algorithms is presented in Figure 8.

### 5.1. Generated Templates

Again, we present the data from each person individually. The main comparison is about the full and Beta frequency bands.

#### 5.1.1. Person 1

Table 4 shows the areas that were activated during the activity using J48 and Random Tree algorithms with “full” band and Beta band for Person 1.

For Person 1, the electrodes that showed significant relevance (i.e., were activated) belong to the following areas (Table 4): temporal, which deals with the perception of biological movements; parietal, which is responsible for the tactile sense; frontal, responsible for executive functions and management of cognitive resources; and occipital, where the image processing takes place. As the subject is a sighted person, the activation of the occipital lobes was expected, based on the hypothesis of the first case study. However, with the application of the J48 algorithm with the Beta band, the occipital lobe was not stimulated at the time of the test.

#### 5.1.2. Person 2

Table 5 shows the areas that were activated during the activity using J48 and Random Tree algorithms with “full” band and Beta band for Person 2.

For Person 2, the electrodes that showed significant relevance (i.e., were activated) belong to the following areas (Table 5): temporal, which deals with the perception of biological movements; parietal, which is responsible for the tactile sense; frontal, responsible for executive functions and management of cognitive resources; and occipital, where the image processing takes place. As the subject is a sighted person, the activation of the occipital lobes was expected, based on the hypothesis of the first case study. However, with the application of the J48 algorithm with the Beta band, the occipital lobe was not stimulated at the time of the test.

#### 5.1.3. Person 3

Table 6 shows the areas that were activated during the activity using J48 and Random Tree algorithms with “full” band and Beta band for Person 3.

For Person 3, the electrodes that showed significant relevance (i.e., were activated) belong to the following areas (Table 6): temporal, which deals with the perception of biological movements; parietal, which is responsible for the tactile sense; frontal, responsible for executive functions and management of cognitive resources; and occipital, where the image processing takes place. As the subject is a blind person, the activation of the parietal lobes was expected, based on the hypothesis of the first case study, and this hypothesis was confirmed.

#### 5.1.4. Person 4

Table 7 shows the areas that were activated during the activity using J48 and Random Tree algorithms with “full” band and Beta band for Person 4.

For Person 4, the electrodes that showed significant relevance (i.e., were activated) belong to the following areas (Table 7): temporal, which deals with the perception of biological movements; parietal, which is responsible for the tactile sense; frontal, responsible for executive functions and management of cognitive resources; and occipital, where the image processing takes place. As the subject is a blind person, the activation of the parietal lobes was expected, based on the hypothesis of the first case study, and this hypothesis was confirmed.

#### 5.1.5. Analysis Data

Table 2 summarizes the classification accuracy for each algorithm applied to full frequency band and Beta frequency band. The second column “J48” with full band presents the data from first case study. The best classification accuracy was obtained applying the Random Tree algorithm to the full frequency band (49.47%). The lower was using J48 with the Beta frequency band.

For Persons 1 and 2, the highest accuracy occurred using Random Tree with the full frequency band. For Person 3, the highest classification accuracy was Random Tree applied to the Beta band. Only for Person 4, the highest accuracy was obtained using J48.

Based in Table 2, only for Person 3, using the Beta band improved the classification accuracy. For all other persons, the highest classification accuracy was obtained using the full frequency band.

## 6. Conclusions and Further Work

This work proposed a methodology of EEG analysis using decision trees. To validate our methodology, Section 4 discusses and analyzes several trees, showing that they can be easy to interpret and, consequently, very useful for interpreting brain signals. Through decision trees, it is easy to see which electrodes have a significant variation, and in how many cases an electrode had a significant variation in each object.

One issue that can be raised is the accuracy of the generated models. The accuracy was 45% in average. This can be considered a low accuracy for a classification algorithm such as the decision tree model, but to allow easy understanding of the results, the output trees cannot be too large. When the learning algorithm was set to create larger trees, it was able to achieve an accuracy rate of 90% or higher, but the output trees were too large and difficult to analyze. Thus, to obtain easily understandable trees, a tree with very specific knowledge is not desirable.

Based on the resulting decision trees, we observed that sighted people had significant activity in the occipital lobe, which is responsible for the sense of vision, even when they were blindfolded. We believe that this happened because they accessed visual memory to aid them in identifying the objects.

However, blind people showed no significant activity in the occipital lobe in the models created by the J48 algorithm. Therefore, our experience suggests that the brains of blind people and people with normal vision have different ways of carrying out spatial activities, even if they are placed under the same conditions, since the sighted test subjects were blindfolded.

Afterwards, we extended the methodology by using two classification algorithms: J48 and Random Tree, and they were applied to different frequency bands, full frequency band and Beta frequency band, to find out which algorithm and which frequency would have the best results. The hypotheses are summarized and discussed here according to the results.

1. Random Tree has higher classification accuracy than J48
2. Both algorithms will get higher classification accuracy using only the Beta frequency band

The results suggest that using Random Tree can improve the classification accuracy of EEG signals, compared to the J48 algorithm. Thus, hypothesis number 1 was confirmed, although not for all cases.

However, using Beta band frequency was no better than using the full band. Therefore, hypothesis number 2 was not confirmed. We supposed that it is because there is a loss of information by ignoring the other bands.

Some further work needs to be done to confirm these outcomes, such as the following:

- To apply other data-mining techniques, such as SVM (Support Vector Machines), clustering, and neural networks to compare classification accuracy; remembering that the outcomes of these techniques are not readable, we will need some kind of method to explain these “Black Box” outcomes [27]
- To apply temporal data-mining techniques, since the collected data is directly related to time [28]
- To apply statistical techniques of multivariate analysis to find out variables correlation, and then select the most relevant variables (electrodes) to analyze

Also, both algorithms have as output a decision tree that represents an algorithm to classify new instances. However, as demonstrated by the first case study, decision trees are very easy to read and to understand, so they can be used not only to classify new instances, but also to easily and quickly analyze what are the most significant electrodes activated during a task.

## Figures and Tables

**Table 1:** Brain regions, electrodes, and main functions, adapted from [10, 11].

Brain region	Electrodes	Proprietary functions
Frontal lobe	Fp1, Fp2, AFz, F7, F3, Fz, F4, F8, FC5, FC1, FCz, FC2, and FC6	Executive functions (management of cognitive/emotional resources in a given task)
Temporal lobe	T7, TP9, T8, and T10	Perception of biological motion
Parietal lobe	P7, P3, Pz, P4, P8, PO9, PO10, CP1, CP2, CP5, and CP6	Somatosensory perception, spatial representations, and tactile perceptions
Occipital lobe	O1, Oz, and O2	View images (including during a dialogue)

**Table 2:** Comparison of classification accuracy between algorithms with full frequency band and only Beta band.

Person	Full band		Only Beta band	
	J48 (%)	Random Tree (%)	J48 (%)	Random Tree (%)
1	46.19	**49.08**	36.34	48.23
2	44.19	**49.47**	44.74	42.70
3	43.00	41.85	37.68	**46.63**
4	**45.54**	45.44	41.73	44.61

**Table 3:** Brain areas that were present in each individual's model. The letter X indicates the areas that presented the channels in the branches of the generated trees, and NDA indicates that the area did not present channels in the branches of the trees of the generated models.

Area	Person 1	Person 2	Person 3	Person 4
Frontal	X	X	X	NDA
Temporal	X	X	X	NDA
Parietal	X	NDA	X	X
Occipital	X	X	NDA	X

**Table 4:** Activated areas of Person 1. *T*  temporal area; *P*  parietal area; *F*  frontal area; *O*  occipital area.

Algorithm	Full band	Beta band
J48	*T*, *P*, *F*, *O*	*T*, *P*, *F*
Random Tree	*P*, *F*, *O*	*P*, *F*, *T*, *O*

**Table 5:** Activated areas of Person 2. *T*  temporal area; *P*  parietal area; *F*  frontal area; *O*  occipital area.

Algorithm	Full band	Beta band
J48	*F*, *T*, *O*, *P*	*P*, *T*, *F*
Random Tree	*P*, *T*, *F*	*F*, *T*, *O*, *P*

**Table 6:** Activated areas of Person 3. T  temporal area; P  parietal area; F  frontal area; O  occipital area.

Algorithm	Full band	Beta band
J48	*T*, *P*, *F*	*F*, *O*, *P*, *T*
Random Tree	*O*, *F*, *T*, *P*	*F*, *O*, *P*

**Table 7:** Activated areas of Person 4. *T*  temporal area; *P*  parietal area; *F*  frontal area; *O*  occipital area.

Algorithm	Full Band	Beta Band
J48	*O*, *P*	*O*, *P*, *F*
Random Tree	*P*, *F*, *O*, *T*	*O*, *P*, *T*, *F*

## Data Availability

All data used to support the findings of this study may be released upon application to the corresponding author (Diana F. Adamatii), who can be contacted via email, dianaada@gmail.com.
